# Mapping interactions between the sustainable development goals: lessons learned and ways forward

**DOI:** 10.1007/s11625-018-0604-z

**Published:** 2018-07-13

**Authors:** Måns Nilsson, Elinor Chisholm, David Griggs, Philippa Howden-Chapman, David McCollum, Peter Messerli, Barbara Neumann, Anne-Sophie Stevance, Martin Visbeck, Mark Stafford-Smith

**Affiliations:** 1 Stockholm Environment Institute, Sweden and Royal Institute of Technology, Stockholm, Sweden; 2 He Kainga Oranga/Housing and Health Research Programme, Department of Public Health, University of Otago, Wellington, New Zealand; 3 Institute for Global Sustainable Development, University of Warwick, Coventry, UK; 4 Monash Sustainable Development Institute, Monash University, Melbourne, Australia; 5 Energy Program at the International Institute for Applied Systems Analysis (IIASA), Laxenburg, Austria; 6 Centre for Development and Environment (CDE), University of Bern, Bern, Switzerland; 7 Institute for Advanced Sustainability Studies (IASS), Potsdam, Germany; 8 International Council for Science (ICSU), Paris, France; 9 GEOMAR Helmholtz Centre for Ocean Research Kiel and Kiel University, Kiel, Germany; 10 CSIRO Land and Water, Canberra, Australia

**Keywords:** 2030 Agenda, Interlinkages, Connections, SDG, Development, Knowledge platform

## Abstract

Pursuing integrated research and decision-making to advance action on the sustainable development goals (SDGs) fundamentally depends on understanding interactions between the SDGs, both negative ones (“trade-offs”) and positive ones (“co-benefits”). This quest, triggered by the 2030 Agenda, has however pointed to a gap in current research and policy analysis regarding how to think systematically about interactions across the SDGs. This paper synthesizes experiences and insights from the application of a new conceptual framework for mapping and assessing SDG interactions using a defined typology and characterization approach. Drawing on results from a major international research study applied to the SDGs on health, energy and the ocean, it analyses how interactions depend on key factors such as geographical context, resource endowments, time horizon and governance. The paper discusses the future potential, barriers and opportunities for applying the approach in scientific research, in policy making and in bridging the two through a global SDG Interactions Knowledge Platform as a key mechanism for assembling, systematizing and aggregating knowledge on interactions.

## Introduction

In these early years of the 2030 Agenda implementation, the quest for informed and integrated decision-making and coherence in policy has become a critical issue amongst both national governments and international organizations (OECD 2016). The so-called “indivisible” agenda partly responded to lessons learned from the MDG process, which had seen problems arising from fragmentation and siloed implementation (Vandemoortele 2011). The 2030 Agenda emphasizes the importance of understanding—and acting upon—interlinkages between policy areas articulated in the sustainable development goals (SDGs). It also emphasizes the importance of partnerships for implementation. The two are clearly connected: actors in governance often represent certain policy issues and objectives, and building partnerships between actors fundamentally depends on understanding what the interactions look like between the policy issues or sectors they represent. On that basis, decision makers can judge who to partner with, and in what ways (Weitz et al. 2017).

Interactions can be both positive and negative. A better grip on *positive* interactions provides the prospect of identifying co-benefits that enable achieving outcomes at lower cost or with enhanced impact, through coordination of action. There is a growing number of such examples. For example, McCollum et al. (2011) showed how simultaneously targeting energy security, air pollution and climate change in energy systems could achieve all three goals at only slightly higher cost than achieving just the climate change goal alone. The WHO has applied a similar approach for leveraging co-benefits between urban air quality, transport, housing, climate change and health (Chapman et al. 2016; WHO 2011). There are also examples of how co-benefits move across scales. For example, Lacey et al. (2017) show that a 20-year global phasing out of solid-fuel cook stoves could reduce global warming by 0.08 °C by 2050 at the same time as avoiding 260,000 premature deaths per year from local pollution impacts. Springmann et al. (2016) show that a transition to plant-based diets could reduce global mortality by 6–10% whilst reducing food-related greenhouse gas emissions by up to 70% in 2050 compared to a reference scenario. Zhang et al. (2015) show how regionally differentiated targets for nitrogen management could help meet food, land degradation and climate targets efficiently.

Also negative interactions must be accounted for. Identifying potential trade-offs enables mitigation and management of conflicts between goals. Rogelj et al. (2013) showed how the potential (small) trade-off between providing ‘energy for all’ and meeting a 2 °C climate change target could be managed by setting specific minimum targets for the rates of change in energy and carbon intensity. At a national level, Gao and Bryan (2017) explore the feasibility of achieving multiple targets in the Australian land use and show that managing trade-offs require targeted action in sectors such as energy, food production and water management. These types of considerations also help to identify ‘winners and losers’ from particular pathways, the understanding (and potential compensation) of whom may be critical for achieving action (cf. urban trade-offs explored by Vargo et al. 2016).

Sometimes, recurrent patterns of interactions among a small set of goals and interactions are referred to as “nexus” issues (e.g. the water-energy-food nexus; Weitz et al. 2014). However, these nexus areas are rarely defined based on a methodical approach (Wichelns 2017). Beyond the few examples above, there is a very large and diverse knowledge base on interactions. An almost indefinite number of such ad hoc examples can be listed, however there is no framework currently in use that supports the aggregation and systematization of this fragmented knowledge. Joined-up assessments of interactions to achieve the SDGs over time are now pursued also through integrated assessment modelling (van Vuuren and Kok 2012) for example in the collaborative project “The World in 2050”, however with data sets for only certain SDGs applicable for this form of analysis.

Hence, approaches for how to more systematically identify, characterize and address interactions between all sustainable development policy issues remains a challenge. Attempts have been made but have also exposed a clear lack of tools and frameworks for doing so (e.g. Griggs et al. 2014). Building the knowledge base around interactions will be important to focus interventions more effectively. Stafford-Smith et al. (2016) have noted that the means of implementation (MOI) (MOIs constitute 42 of the targets in SDG17 as well as the “alphabetic” targets under each of the other SDGs) could themselves be activated in more-or-less integrated ways. Systematically focusing the MOI (finance, technology, capacity building, trade, policy coherence, partnerships, data, monitoring and accountability) on SDG interactions can lead to more integrated decision-making and coherent policy approaches.

This paper aims to articulate a way forward for accumulating a global knowledge base on interactions, using lessons from a pilot application of a novel framework to map interactions between the SDGs (Nilsson et al. 2016; ICSU 2017). We briefly introduce the (previously published) SDG interactions framework in “Framework and method”. We then present some examples of results, with a focus on the role of context variables in three selected case domains where the framework has been applied (“Results”). In “Discussion: using the SDG interactions framework in policy and in research”, we discuss the potential of this approach to interactions for informing (1) more integrated policy and implementation for international and national/subnational processes), and (2) scientific research processes. Finally, we discuss how the approach could be developed into a knowledge platform that systematizes and aggregates diverse knowledge (also section “Discussion: using the SDG interactions framework in policy and in research”).

The approach taken is here applied specifically to the SDGs, although it is in principle applicable for assessment of interactions across any policy areas.

## Framework and method

In 2016, an international research initiative was taken to advance the conceptual and empirical basis for understanding interactions. The SDG interactions framework was developed (Nilsson et al. 2016) and subsequently applied in a multi-team study with a focus on the SDGs on food (SDG 2), health (SDG 3), energy (SDG 7) and oceans (SDG 14; ICSU 2017) (this selection of areas was determined by the International Council for Science (ICSU) and was depending on the availability of research teams). The core of the framework is a typology and scoring of interactions on a 7-point scale (Table 1). The typology characterizes the nature of binary relationships between SDGs at the target level, i.e. between progress on one SDG target and progress (or not) on another.^1^

**Table 1:** Seven types of interactions between SDG targets (Nilsson et al. 2016)

Interaction label	Meaning
+3 Indivisible	Progress on one target automatically delivers progress on another
+2 Reinforcing	Progress on one target makes it easier to make progress on another
+1 Enabling	Progress on one target creates conditions that enable progress on another
±0 Consistent	There is no significant link between two targets’ progress
−1 Constraining	Progress on one target constrains the options for how to deliver on another
−2 Counteracting	Progress on one target makes it more difficult to make progress on another
−3 Cancelling	Progress on one target automatically leads to a negative impact on another

The framework emphasizes the importance of assessing interactions with a view to key contextual determinants and influencers of the interaction, including the governance and geographical contexts, implementation technologies and policies and time-horizon. The framework also emphasizes the directionality of interactions since interactions are either uni-directional (such as between electricity access and education) or bi-directional but asymmetric (such as between health and agricultural productivity). Interactions can create positive or negative feedback loops, examples of which are visible in resource areas such as ocean fisheries, where enhanced incomes lead to increasing investments in improved gear, which in turn lead to growing incomes, ultimately putting the resource base at risk.

Apart from the ICSU (2017) study, the framework has been tested using a systems approach at national scale (Weitz et al. 2017). The latter study scored pair-wise target interactions in a cross-impact matrix of 34 × 34 SDG target interactions using the 7-point scale. Having completed the matrix of binary relations it used network theories and systems analysis to derive information about which targets are the most influential on other targets, either positively or negatively, and used algorithms to identify clusters of targets across the 2030 Agenda. Other empirical efforts to use network analysis for assessing SDG interactions include recent work by Zhou and Moinuddin (2017).

In parallel to the research-based application, which is the focus of this paper, the SDG interactions framework has gained attention and interest in national and international policy venues. The benefits of using interactions thinking to better navigate in the multiple dimensions of the 2030 Agenda and identifying some areas of focus, have been noted. It has been applied in studies with United Nations (Nilsson 2017) and at the High-Level Political Forum (HLPF) with the OECD’s policy coherence team, and it has made its way into tools and methods guidelines within the UN system (United Nations Development Group 2017). The approach has also been applied with UNDP and the governments of Sri Lanka and Mongolia during 2017–2018.

At the same time, the framework has generated various critiques (not yet published as far as we have seen). The framework typically triggers questions regarding the assumptions that go into assessing the interaction. As noted above and in Nilsson et al. (2016), interactions depend on context variables, and assumptions about them will determine the interaction. In our view, this does not render the framework moot, but it makes it even more important that analysts are transparent about their assumptions and critically review the assessment process and lessons learned.

The framework was applied through literature reviews carried out by expert teams. The approach was a two-stage process. The first step entailed expert identification, to develop a diverse knowledge base possessed by the teams and using syntheses and assessment reports as entry points for issue identification. The second step entailed systematic online searches in academic library databases to establish the evidence base for important issues. Further details on methodologies applied are elaborated in McCollum et al. (2018).

## Results

This section synthesizes experience from applying the interactions framework. For a full account of results see ICSU (2017) and (for energy) McCollum et al. 2018). The focus here is on the importance of contextual factors and conditions that could influence the nature of the interaction between two SDG targets (Nilsson et al. 2016). Below, we discuss, with examples from the different SDG areas, to what extent and how these key conditions have materialized in the empirical studies, drawn from Howden-Chapman et al. (2017), Schmidt et al. (2017), and McCollum et al. (2017). We focus the discussion here on the governance context, the geographical context and the time horizon (identified in Nilsson et al. (2016) as the most critical context variables). The examples provided are not meant to be representative across all SDG target interactions but have been selected because they usefully illustrate how the contextual factors and conditions affect the assessment.

### Governance context

What governance approach one uses to achieve a target, and within what institutional context this happens, can influence the character of the interaction. How sensitive has the assessment been to assumptions about governance and institutions?

Progress on any goal is often likely to support *health*. For example, gender equality (SDG 5) support greater reproductive health (Wang 2010). Clean water (SDG 6) and climate action (SDG 13) will reduce the spread of infectious disease (Watts et al. 2018; UNICEF and WHO 2015; Bain et al. 2014; WHO 2011) Progress on clean energy (SDG 7) will improve respiratory health (WHO 2014a, b). However, these assessments are highly dependent on governance, and a positive interaction can be reversed in the absence of appropriate governance measures. Increasing agricultural productivity, which is a key target for ending hunger (SDG 2), enables nutrition by increasing incomes (Byerlee et al. 2005), which supports several health targets (FAO et al. 2017). Yet, without research and monitoring to ensure that agricultural expansion does not have adverse effects on the environment, this can lead to ecosystem shifts with negative health outcomes. For instance, using insecticides in agriculture is associated with higher resistance in malaria vector mosquito populations (Reid and McKenzie 2016), and irrigation and other agricultural practices can create new habitats for vectors of malaria and other diseases (World Bank 2008). Governments can mitigate these risks to human health through measures such as assessing connections between agriculture, water use and infectious diseases in different places, linking health, veterinary and wildlife surveillance systems, and developing community-based vector-borne disease control models (WHO 2013). Stable institutions, and the resources to carry out research, and implement and enforce regulation, are necessary to reduce the risks associated with increasing agricultural productivity. Governance arrangements are therefore crucial to ensuring that expanding agricultural systems supports health.

An example drawn from *energy* is how the distributional impacts of new energy policies (e.g., supporting renewables and energy efficiency) are dependent on instrument design, and if these costs fall disproportionately on the poor, then this could impair progress toward universal energy access and, by extension, counteract the fight to eliminate poverty (SDG 1) (Sovacool et al. 2016). Similarly, the design of regulatory mandates will greatly affect the nature of interactions with other SDGs. For instance, whether countries and cities choose to meet the energy efficiency and renewable energy targets by mandating electric vehicles, regulating household heating technology, or requiring that biofuels be blended into the fuel mix; this choice will have varying impacts on outdoor and indoor air quality and human health (SDGs 3 and 11), food and ecosystems (SDG 2), and jobs and innovation (SDG 8) (McCollum et al. 2018).

On *oceans*, interactions between SDG targets are often dependent on policy design or governance regimes, and on the measures taken to achieve a specific target (Schmidt et al. 2017). For example, the creation of marine protected areas (MPAs) as promoted under target 14.5, is generally seen as a powerful and effective instrument to protect, conserve and restore coastal and marine ecosystems, species and habitats, and to increase species richness and biodiversity (OECD 2017). Providing various co-benefits to fisheries and livelihoods of coastal communities (Agardy 2000; Bennett and Dearden 2014; Fisher and Christopher 2007), MPAs have a range of positive effects on other areas of sustainable development by enabling progress on poverty eradication (SDG 1) and food security (SDG 2) (Schmidt et al. 2017, p 178 and p 184 ff). However, the success and strength of these co-benefits of MPAs depend on how, and how well, the respective MPAs are managed, and on policy integration across sectors (Edgar et al. 2014; Bennett and Dearden 2014; Spalding et al. 2016). Some MPAs are no-take zones and thus optimally providing breeding grounds for fish, while others still allow commercial fishing. Moreover, the effectiveness of protection measures is vastly different between nations: they often suffer from lack of transparent and effective governance and/or weak enforcement. Thus, MPAs may also limit access to, and create competition for, resources and thus impede the goals addressing hunger and poverty if they are not managed in accordance with other sectors (Mascia et al. 2010; Singh et al. 2017; Schmidt et al. 2017, p 184 ff), especially in the short term and depending on the conservation status applied. In the worst case, inadequate governance could even turn a positive interaction into a negative one.

### Geographical context

A second key dimension that shapes the interaction is the geographical place and scale (and the resources available there). In the *health* area, interactions were highly dependent on geographical place and scale. As noted already, health targets can be supported by increasing agricultural productivity (SDG 2) as it increases people’s incomes. Yet, in some areas, expanding the land used for agricultural production may induce crossover of zoonotic pathogens from life-stock to humans, or expose people to increased malaria (Arrow et al. 2004). Geographical place is also likely to determine to what extent promoting gender equality (SDG 5) supports health. In this case, it is likely that the principal reinforcing interaction is generally valid, but the strength of it depends on where it takes place, and the level of equality you start from in this place. Improving gender equality generally enables the achievement of better health, but the interaction will be stronger where women face greater inequalities. In contexts where inequalities are great, women’s health issues are often under-prioritized and under-funded; and progress on equality can then lead to overall improved health. Health gains may be immediate, as when they directly improve resources or access for women, or long-term are mediated through child care (WHO 2016; Leach 2016).

For *energy*, geographical context influences how interactions play out. For example, the expansion of renewables in Sweden may include hydropower as an option, which interacts strongly with freshwater ecosystems (SDG 6). In Denmark, on the other hand, hydropower is not an option, but wind power (both onshore and offshore) is, which interacts with terrestrial and ocean systems (SDGs 15 and 14) (Schwanitz et al. 2017). Thus, interactions with target 7.2 could be with entirely different goal areas in these neighboring countries. Similarly, a country (or part of a country) that is already well-positioned to expand its renewable technology production capacity will stand to gain more from a global expansion of renewables (in terms of jobs, SDG 8) than a region where no such capacity exists (either in terms of technical or human capital) (Babiker and Eckaus 2007; Borenstein 2012). Moreover, in some cases interactions manifest themselves across geographies and across scales. Climate change, for example, is a global problem; hence, the greenhouse gas emissions reductions resulting from renewable energy expansion (target 7.2) in any part of the world will be indivisible from progress on SDG 13 globally. Meanwhile, the air pollutant emissions reductions (part of SDG 3 and SDG 11) brought about by those same strategies will typically be localized, with the benefits accruing mainly to those living in cities in rapidly developing and transition countries (IEA 2016).

For the *ocean*, the geographical context is highly decisive but also difficult to grasp. This is due, on the one hand, to most interactions playing out in specific places such as coastal settings, with spill-over effects for example through trading of marine resources and products or through marine pollution from land-based sources (Schmidt et al. 2017; Newton et al. 2012; Stojanovic and Farmer 2013). On the other hand, processes, activities and impacts in the marine environment evade concrete geographical boundaries due to the transboundary nature of impacts, effects and the marine environment as such (Kavanaugh et al. 2016; Levin et al. 2017). This, and the connectedness between land and sea, complicate the assessment. There also exist global-level linkages such as with SDG 13 (climate action), where cross-scale and cross-geographical interactions become manifest (Schmidt et al. 2017, p 206 ff). Climate change and related effects such as ocean acidification, which is addressed in target 14.3, is a global phenomenon (Pörtner et al. 2014; Wong et al. 2014). The actual impacts such as coral bleaching, though, occur locally or regionally and may cause development problems for coastal communities (Cinner et al. 2012), not to speak of the environmental degradation of specific habitats. Minimizing ocean acidification will likely have beneficial effects on fish stocks (Speers et al. 2016; Olsen et al. 2018) and hence improve livelihoods (SDG 1) and nutrition (SDG 2), especially in developing coastal states where large coastal populations depend on fish as the major source of protein. Moreover, for many developing coastal states and small island developing states (SIDS), coastal and marine tourism is a major economic factor (UNDESA 2015; UNEP 2009). Another example illustrating the importance of geographical context is target 14.1 about preventing and reducing marine pollution. Many coastal areas including remote and even unpopulated oceanic islands have a problem with pollution, for example with plastics, in their coastal zones (Hidalgo-Ruz and Thiel 2013; Lavers and Bond 2017). However, the actual source of this pollution is often located far away and stems from different countries or even regions, owing to the ocean currents. Thus, tackling of plastic pollution, e.g., by investing in sustainable consumption and production (SDG 12) will interact with other SDGs beyond the actual geographical context of its occurrence, requiring regional and global cooperation and agreements on the reduction of marine litter to effectively meet target 14.1.

### Time horizon

Implementation of interventions has different time scales, and progress (or regress) on targets become manifest and detectable on different time scales. Therefore, across the three areas, interactions varied also depending on the time frame applied in the assessment.

Most interactions with *health* differ depending on whether examined on a short-, medium- or long- term. For example, heath goals are supported by access to energy (SDG 7) through its contribution to economic development, and through enabling people to heat or cool their homes, use lighting and cooking facilities, and access services like health care and transport—positive interactions that unfold over multiple years. In the short term, unsafe energy sources such as burning solid fuels exposes people to pollutants that harm respiratory health (WHO 2014a, b, c). In the medium- to long-term, greenhouse gas emissions associated with the use of fossil fuels will accelerate climate change, which affects proximal and distal factors for a wide range of health outcome. Climate change, over the longer term, is expected to cause increased mortality in many regions, due to heat exposure, flooding, diarrheal diseases, malaria, and under-nutrition (WHO 2014b).

For *energy*, time horizons are critical given that energy infrastructures take a long time to set up and are long-lived investments. The demand for these technologies, once built, can persist far into the future (Goldthau 2014). Furthermore, a unit of carbon released into the atmosphere by the energy system between now and 2030 will still be there generating global warming in the next century and beyond. Yet, while energy system change is a decades-long process, near-term and immediate actions promoting renewables and boosting energy efficiency will have short-term positive interactions with e.g. health (see above), employment (SDG 8) and innovation and industrialization goals (SDG 9) (Bhattacharyya et al. 2016).

For the *ocean*, time is both a critical and difficult variable, and many interactions play out over long time horizons. A well-known example of shifting interactions over time can be found in fisheries, which are important for securing food (SDG 2) and livelihoods (SDG 1) in low-income coastal areas. In the short run, improved fishing gear, better access to markets and increasing fishing activity, possibly supported by investments enabling market access, will lead to a reduction of hunger and improved livelihoods. With time, however, fish stocks are at risk of becoming overused and the same effort leading to less and less yield unless sustainable management rules are put in place (e.g. 14.2). Restoring natural resources and ecosystems in a way that they can deliver the desired services, i.e. support achieving other interlinked goals such as SDGs 1, 2, 8 or 13, requires time. But specifying the actual time needed to achieve a desired status is difficult and depends on complex systems dynamics in the ocean, and requires increased scientific and interdisciplinary research, knowledge exchange and technology transfer (see below).

### Overall pattern of positive and negative interactions

Reviewing the interactions assessments reported in ICSU (2017), which examined in total 316 interactions connected to SDG 2, 3, 7 and 14, it transpires that around 80% of the interactions examined were in fact positive, and ca 20% were negative—slightly more for in the case of oceans, and less for health and energy goals. The -3 (cancelling) type only appears once. Note however that the ICSU study did not conduct a systematic assessment of SDG target interactions but focused on key interactions between selected SDGs and their targets which were identified from an expert-based assessment of interactions at goal level (ICSU 2017). As a consequence, neutral relations were mostly filtered out by design. The sample of three SDGs covered in this study may be more prone to positive interactions than another sample, since the sample was not drawn to be representative. Furthermore, this application of the framework has not considered cross-border impacts that sometimes are negative, such that, for example investments in energy systems in one country might have impacts in another. Weitz et al. (2017), who drew on a more comprehensive sample of targets examining over 1000 interactions, assessed over 50% of interactions as neutral, and less than 5% as negative. This study pulled a sample of the two most relevant targets (determined by the authors) for each SDG in the context of Sweden, and asked the question for each of the 34 targets against each other: “if we make progress on A, how does it affect our ability to make progress on B”?

### Knowledge gaps and research issues

What are the major empirical research and data gaps when it comes to interactions? Which areas are we fairly certain about and which ones are highly uncertain? The account below is not comprehensive but highlights a few areas of notice.

For *health*, it is well known that every SDG connects to SDG 3 in some way: for example, enabling more access to clean water will undoubtedly reduce child mortality and infectious disease (Blomstedt et al. 2018). The evidence that some goals and a number of different targets within SDG 3 support health outcomes is strong. However, more research will be needed to strengthen the evidence of connections with other goals and targets; for example, the connection between air pollution and maternal health, and other non-respiratory health outcomes, is only beginning to become clear (Hu et al. 2014; Malley et al. 2017). Further case-by-case analysis is required for determining how, for example, intensifying agricultural production will affect the environment, including through the expansion of pathogen habitats and the degradation of waterways, as these will have important effects on health.

For *energy*, there is good agreement in the literature that ensuring energy access for the poor, deploying renewables at scale, and boosting energy efficiency will have positive impacts on—and will themselves be aided by—the targets for reduced climate change, achieving poverty alleviation, water availability and quality, human health, natural resources protection, and improved cities (Nerini et al. 2018). On the other hand, there are knowledge gaps for how SDG 7 will interact with labor markets (SDG 8), inequalities (SDGs 5 and 10) or oceans (SDG 14) (McCollum et al. 2018).

For the *ocean*, knowledge gaps are large in relation to all interactions. These gaps have various causes and are often specific to the issues of marine resources, and some cannot be immediately addressed with more research. Many relate to a lack of and restricted access to knowledge, data or information, and a lack of standardized data collection protocols—and compliance with them. For example, under- and misreporting of landings of fish catches and lack of stock assessments, occurring not exclusively but largely in artisanal fisheries and low-income countries, is a big problem (Pörtner et al. 2014). Not knowing the resource base and the actual catches hampers the achievement of targets that address sustainable fisheries and the sustainable management and protection of marine and coastal ecosystems, as well as other SDGs such as SDG 2. Additional knowledge gaps refer to the concrete relations between marine conservation and human well-being, economic or social development, or climate change, and how these change (Agardy 2000; Agardy et al. 2005). Finally, insufficient coordination across political and sectoral boundaries, and limitations in capacity for data analysis or mainstreaming into policies is a major reason for knowledge gaps, especially in developing countries.

## Discussion: using the SDG interactions framework in policy and in research

The early experiences using the SDG interactions framework, as presented above, suggest that it can play a role in supporting the structuring of a science-policy interface on the SDGs, in part by systematizing policy-relevant and useable knowledge and in part by inducing joined-up learning and dialogue across different sector and stakeholders in policy and planning. To support policy efforts at appropriate levels of decision-making, the contextual dimensions must be front and center of the assessment process. The framework can also support priority setting in research such that new funding could be oriented to identified knowledge gaps related to interactions to and from each SDG area. Below we discuss separately potential uses of the framework, first in policy and planning, and then in science. After this, we present a preliminary design of a knowledge platform that could support both these uses.

### Policy and planning uses

The initial applications of the framework demonstrate potential as a tool for policy dialogues and learning (as noted earlier, it has already been used in policy workshops at national and international levels). First, through the deployment of a simple and intuitive conceptual language, the framework enables engagement of policy makers with varying levels of seniority and technical expertise. Second, the juxtaposition of different policy sectors forces engagement and debate across government departments, something that in many jurisdictions is very rare. Third, by providing a common language and template for discussion, the interactions framework enables aggregation of systematic lessons and insights regarding co-benefits and trade-offs that need to be observed.

Here, context-specific case studies of interactions can be collected and the knowledge about the character of interactions that they represent can be coded using the 7-point scale, together with records of the contextual dimensions (such as time, geography, governance). Over time this collection can be lead to syntheses with a growing understanding of how to manage the interactions to best effect should help set priorities for policy interventions through the various mechanisms implied by the means of implementation.

At the global level, a systematic synthesis of interactions would help the UN review process (e.g. through its High Level Political Forum (HLPF)) identify areas of opportunity (co-benefits) or contention (trade-offs) towards which to steer global negotiations. At the national level, the knowledge generated and systematized through the approach is sometimes directly applicable into policy and planning processes such as the national strategies or plans for implementing the SDGs. At the local level, the assessment can become very specific and generate knowledge on what interactions may provide the most co-benefits and how to resolve trade-offs in delivering SDGs locally.

This knowledge can be impactful in decision-making at all levels by demonstrating that there are many co-benefits that are not normally considered when isolated sectoral perspectives are pursued. It also helps characterising the challenges of negative interactions and identifying ways to mitigate them. Finally, with a quantitative scoring scale, collected data can be coded and analysed for different systemic properties using decision support software. It is then possible to identify coherent clusters of targets that can more easily be pursued together (Weitz et al. 2017).

### Scientific research uses

While the SDG interactions framework has considerable potential as a policy tool, it can also be used in scientific research. First it can be used as a framework for literature surveys and knowledge data bases by coding published case studies and empirical data sets so they can be accessed by scholars and students interested in a particular interaction related to a specific SDG or target in a selected geographical context. Such a collation could look not only at “diagnostics” i.e. empirical observation of interactions, but also at documented actions and solutions that deliver co-benefits or mitigate trade-offs. This could become an experientially-based compendium of case studies from which to drive implementation and prioritization.

In addition, the framework can support the framing of global or regional syntheses, analyses and modelling efforts to help identify the total emergent value of tackling specific interactions in a coordinated way. This would help make the case for more coordination in global negotiations and perhaps identify some trade-offs that need particular attention at global level, as identified in the planetary health approach (Whitmee et al. 2015). It could also be linked to integrated assessment modelling as currently under way with the “The World in 2050” initiative and introduce a systemic procedure for developing and analyzing sustainable development scenarios.

Finally, the framework can be used for identifying research needs and for prioritizing research where funding is needed, either due to important knowledge gaps; or by shedding light on particularly thorny, ambiguous and critical interactions. Discussing the knowledge base, and where the gaps are, it becomes clear that this is a question with many facets. For some interactions we may have excellent, multiple case-study knowledge but generalization may be difficult with quantitative and statistical data lacking. For others, we may have general statistics but difficulty understanding how interaction play out in context. Finally, there might be quantitative models for understanding interactions between climate change and food production at the global level or for the European Union, but for certain regions such as Africa, data gaps make modelling very coarse.

### An SDG interactions knowledge platform

As demonstrated above, the SDG interactions framework could play a role in influencing both implementation policies and science priority setting at national and global levels; ideally, it would be a tool and a language for learning and dialogue between science, society and policy. Today, the knowledge base and its use locally, in science, and in policy is limited, due to lack of organization, systematization and aggregation. Resolving this would require developing a knowledge platform that collates information on interactions coherently in a single place (Nilsson 2017). This will help to support the implementation of the 2030 Agenda in three significant ways:

1. A key lever for innovative pathways to sustainability lies in harnessing the co-benefits between different SDGs and their targets. Yet, information about interactions is poorly documented and fragmented across the specialized disciplines and sectors involved in different SDGs. Besides the undeniable knowledge gaps, even accessing existing knowledge is a major problem for timely insights into policy and planning decisions. It is essential that the different forms of knowledge and evidence are systematically accumulated and become openly accessible and useable to science and different stakeholders.
2. As seen above, context matters when defining what sustainable development means and what pathways may enable it. Yet, in an increasingly connected world, the transformative potential of the 2030 Agenda lies in its universality, where essential leverage points for change in a given context may be found in a different place or at another spatial scale. A global knowledge platform is hence important not only to understand the diversity of sustainability solutions needed and to exchange experiences and good practices, but more importantly also to explore how such solutions reinforce or contradict each other across different places, regions and scales.
3. Sharing and exchanging knowledge allows an understanding of who is involved in both science and policy-making spheres. A knowledge platform on SDG interactions is hence also a means to initiate new collaborations between actors across science, policy, local communities and the private sector. As highlighted by SDG 17, such well-targeted partnerships for change are important to induce step-change improvements in development.

**Fig. 1:**
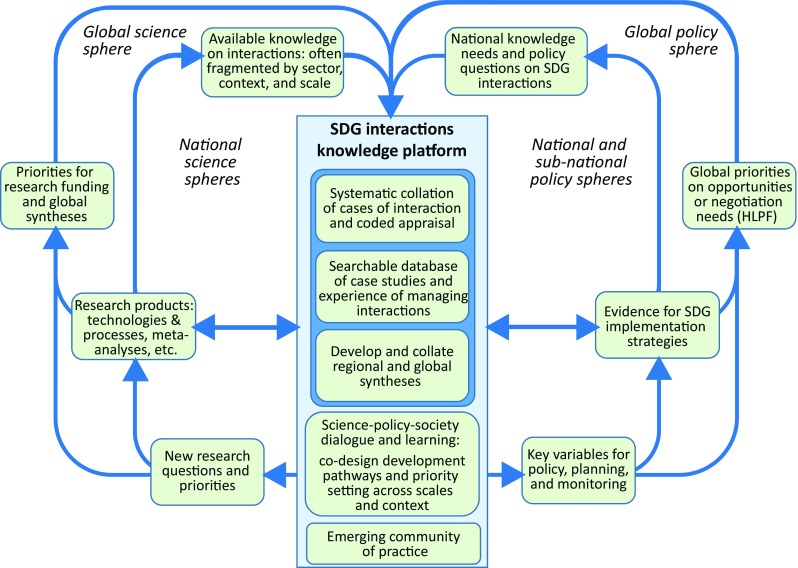
Proposed components of a web-based Knowledge Platform on SDG Interactions and processes of knowledge use in the science and policy spheres, showing the core collation of case studies coded in a way that they can be searched, matched and synthesized, and thereby inform stakeholder dialogue and learning in a developing community of practice. The outer cycles show how this information could flow (right) through local implementation and global policy making, and (left) into driving national or global level research, generally in a co-designed way

Based on these three needs we propose a knowledge platform to help bring together science and policy actors in their use of knowledge on SDG interactions globally (Fig. 1). Initially this would be a curated web-based knowledge repository, but we envisage that it would gradually become a more interactive learning and collaboration site, and eventually a platform for a global community of practice about SDG interactions. Although a number of extant websites aim to collate case studies related to the SDGs, none of these focus on interactions, nor are they structured to identify benefits from managing synergies and trade-offs.^2^ This platform would advance the accumulation of an increasingly comprehensive body of evidence over time (based on an understanding of comparable local contexts) and support syntheses that integrate the case studies, thereby enabling the sharing of knowledge and experiences among different partners, and facilitating joint learning processes to innovate development pathways in specific contexts but also to generalize and set priorities across contexts and scales.

It is worth noting that this is not intended to be a formalized large-scale assessment process like the Intergovernmental Panel on Climate Change or the Intergovernmental Panel on Biodiversity and Ecosystem Services, although the policy learning that emerges should assist the High Level Political Forum that reviews progress on the 2030 Agenda, for example by providing source material for the regular Global Sustainable Development Reports (GSDR).

To deliver to the needs above, at the core of the platform (Fig. 1) is a systemic collation of case studies describing interactions, which can then be searched by stakeholders seeking to learn from the experience of others as well as used to develop higher order syntheses. Both of these uses require that the case studies be systematically coded in terms of both the interactions they address and the geographical, governance or technological context to which the knowledge refers. The initial minimum features of such a protocol are proposed in Table 2, acknowledging that these will be improved with initial testing and use. It will be apparent that this protocol learns directly from the experiences of applying the SDG interactions framework as illustrated in section “Results” above: it includes (aspects (ii)–(iv)) the geographic and governance context of the case study, its spatial and temporal dimensions, and the nature of its interactions as classified by the SDG interactions framework.

**Table 2:** Protocol for a systematic collation of cases of interaction and their appraisal

General aspects	Detailed features
(i) Knowledge source	Authors, year and title of publication Type of source (peer-reviewed, grey literature, reports, etc.)
(ii) Context of knowledge claim	Geographical place, country, or region Spatial scale(s) from local to global Coding time frame in which the interaction manifests Differential short and long-term effects Irreversibility
(iii) Type of interaction	Goals or targets interacting in the case study Directionality of interaction
(iv) Characteristics of interaction (as the platform learns over time, this may become a more explicit classification)	Generalized appraisal using the 7-point scale Brief description of processes studied and data supporting the generalized appraisal Notes on the social context of the processes studied and the role of governance
(v) Trade-offs and co-benefits	Account of key trade-offs or co-benefits The winning stakeholders The losing stakeholders Any quantifiable facts and figures
(vi) Management and development experiences	Transformative actions taken to mitigate trade-offs or maximize co-benefits Outcomes and experiences of such measures (quantified where possible) Links to further materials such as additional stories, media reports, etc.

These coding aspects provide the basis for structured database searches: researcher, planners, and policy-makers, at national or sub-national level, will be able to retrieve knowledge on case studies classified by context, scales, and region or country. As the collection grows, it will be possible to identify major knowledge gaps in terms of types of interactions, contexts or scales. The coding protocol (Table 2) also seeks to record quantitative outcomes (aspects (v) and (vi)), that, with their context definition, would enable future syntheses to be undertaken across case studies more easily than the modelling studies cited earlier (e.g. McCollum et al. 2011; Zhang et al. 2015; Springmann et al. 2016; Lacey et al. 2017). Even without such modelling, observing interacting sets of targets across different contextual factors could reveal, for example, how co-benefits at a national level may turn into trade-offs at local level, or how synergistic SDG implementation strategies in Subsaharan Africa may not apply to South Asia. Taken together, the synthesis efforts will aim to provide insights for higher-level strategic questions: where do transformation pathways converge across regions and scale and represent opportunities for up-scaling of strategies, and where do we see contention and the need for negotiation at a global scale?

A simple example is provided by the issue of households burning solid fuels for cooking mentioned in the introduction (Lacey et al. 2017). Local case studies had shown the health benefits of replacing solid fuels with modern power (potentially informing local implementation, Fig. 1 inner right cycle), but the global synthesis was needed to recognize how this could add up to globally significant outcomes in terms of deaths and CO_2_ emissions avoided (helping drive global research activities, Fig. 1 outer left cycle). This identified the regions where prioritizing ‘modern energy for all’ would achieve the greatest global leverage (informing global negotiations, Fig. 1 outer right cycle), but implementation in turn needs to be sensitive to local cultural and technological context (which may trigger further local research, Fig. 1 inner left cycle). This example preceded the platform, but in fact quantifying other possible interaction benefits arising from these interventions (such as improving gender equality, freeing up time for children’s education, and reducing the impact of fuel collection on forests) could now be supported by the platform.

Developing from this core of case study collation, search and synthesis, the platform should aspire to support science-policy-society dialogue and learning. By including ‘who’ in the coding protocol (Table 2, aspects (i) and (v)), it will be possible to identify stakeholders who might come together to discuss a specific context or interaction (or conflict). By recording the outcomes of actions taken (aspect (vi)) to maximize co-benefits and mitigate trade-offs, the platform will support users in the design of SDG implementation strategies.

A knowledge platform on SDG interactions should not be about prescribing courses of action in given contexts. These are political decisions that should emerge through national processes. Hence, eventually, the knowledge platform should proactively support evidence-informed dialogue and learning among different stakeholders. There is an increasing understanding of how to operate such multi-stakeholder activities successfully (e.g. Galafassi et al. 2017; Faling et al. 2017; Butler et al. 2016; Frantzeskaki and Kabisch 2016). From such activity, a community of practice of researchers and societal actors studying and solving SDG interaction challenges could emerge. They might choose to hold conferences, education sessions and outreach events at national, regional or global levels, and thus help to build much needed institutional capacity around the world. As with all such platforms and international assessment processes, it is important to think through risks of losing momentum or lack of incentive amongst users, and how to mitigate them.

## Conclusion

Although the sample of interactions has not been systematic, considering also the results in Weitz et al. (2017), it appears that negative interactions are likely outnumbered by positive ones. This carries an important message to policy makers: if they look outside the priorities of their sectoral turf and at how they influence -and are influenced by—others, they are likely to find common interests and (unexpected) alliances and that more integrated policy making is likely to pay off in terms of more effective development outcomes. Grey reports related to SDGs support this tentative result (PWC 2016). However, it is possible that the sample of SDGs here is not entirely representative in this regard, with a balance more towards positive interactions than some other SDGs, such as goals on economic growth and employment, or land ecosystems.

The pilot application has demonstrated the difficulty in identifying and assessing all key interactions comprehensively. The number of potentially relevant interactions easily becomes overwhelming. Therefore, the selection of targets for analysis is a critical step that needs considerable attention, with input from both political processes and from science.

This initial application of the SDG interactions framework has confirmed how important context-specific understandings are. This conclusion aligns with the overarching premise of the 2030 Agenda with its emphasis on nationally adapted interpretations and action on the SDGs. The natural resource base and geographical context, governance context and socio-cultural conditions play important roles. Often, interactions are generally valid, such as between gender equality and health outcomes, but even in those cases the interaction might differ in practice as impacts will be more visible, and gains more readily made, in places where the starting point is low: a manifestation of diminishing marginal returns as we reach higher levels of progress.

Another conclusion related to context-specificity is the actual meaning of an SDG target in context, a question which has not yet taken center stage in national implementation discourses—where planners have often jumped straight to indicator systems for monitoring progress. This is problematic, because while the initial preparation of the SDG interactions framework (Nilsson et al. 2016) did not touch explicitly on target interpretation, this emerges as a key initial step: before assessing interactions, one needs to articulate what progress on a target means in the (subnational or national) context of implementation in terms of actual, observable outcomes. This interpretation will, in turn, have an impact on the nature of the interactions that are borne out for other SDGs. For example, in the case of health, a target and what actions it might prompt, must be interpreted rather differently depending on the national context, for example depending on what are the most important burdens of disease for the most socio-economically disadvantaged sub-populations.

While the applications so far have been generic or national-level, this experience suggests that there would be value in applying the framework at the local scale—a scale at which many interactions become very tangible and concrete, and the contextual factors become clear. Applying the interactions framework at local scale could dock into ongoing initiatives taken by many subnational regions and cities around the world to use the SDGs as a framework for planning, such as Melbourne (Australia), Baltimore (USA), and Guanajato (Mexico).

This paper has reported on initial lessons regarding how to apply an interaction approach to SDGs in practice. There remain many open questions from a more technical point of view, e.g. how to bring different academic disciplines to the table and generate a common knowledge base for the assessment; how to select the “key” interactions from all possible alternatives; how to tap into statistical data sources; and how to gauge or “calibrate” the different experts’ estimates and characterizations of interactions. These are currently limitations in the the framework that requires attention. We maintain, in this, that the assessment of interactions is not a purely technical exercise: it contains both analytical and socio-political dimensions. The opportunity lies in the flexibility in terms of data availability, such that the framework leads into a kind of analytic-deliberative hybrid process (Renn 1999) entailing both formal evidence and hard data, expert judgment, and stakeholder-driven deliberative data generation. It also establishes some basic principles for a useable global knowledge platform (including a data-base) that assembles how we understand interactions in context with a view to provide better knowledge for coherent SDG implementation.

## Footnotes

1 The 2030 Agenda lists 17 SDGs and, under them, 169 targets.

2 There are many activities focused on a particular interaction but not systematically collating interaction case studies (e.g. UNDP–UNEP Poverty-Environment Initiative, Partnership for Environment and Disaster Risk Reduction, ICSU’s programme on Urban Health and Well-being, UNEP’s Gender and the Environment); there are a few sites collating case studies, but these are not generally focused on interactions or are not taking a systematic approach to the case studies (e.g. Partnerships for SDGs online platform, UNEP SDG Synergies portal). Connecting material across these types of sites is one source for this platform.
